# Supplementary material for: Prioritization of New Candidate Genes for Rare Genetic Diseases by a Disease-Aware Evaluation of Heterogeneous Molecular Networks
Source: Int J Mol Sci. 2023 Jan 14;24(2):1661. doi: 10.3390/ijms24021661 (PMC9864172; doi:10.3390/ijms24021661)
Supplement: Supplementary file 1 [file ijms-24-01661-s001.zip › ijms-2031959-supplementary.pdf]

Supplementary Information

Prioritization of new candidate genes for rare genetic diseases by a disease-aware evaluation of heterogeneous molecular networks

Lorena de la Fuente[1,2,3], Marta Del Pozo-Valero[1,2], Irene Perea-Romero[1,2], Fiona Blanco-Kelly[1,2], Lidia Fernández-Caballero[1,2], Marta Cortón[1,2], Carmen Ayuso[1,2], Pablo Mínguez[1,2,3#]

[1]Department of Genetics, Health Research Institute–Fundación Jiménez Díaz University Hospital, Universidad Autónoma de Madrid (IIS-FJD, UAM), 28049 Madrid, Spain

[2]Center for Biomedical Network Research on Rare Diseases (CIBERER), Instituto de Salud Carlos III (ISCIII), 28040 Madrid, Spain

[3]Bioinformatics Unit, Health Research Institute–Fundación Jiménez Díaz University Hospital, Universidad Autónoma de Madrid (IIS-FJD, UAM), 28049 Madrid, Spain

[#]Corresponding author: Pablo Minguez (pablo.minguez@quironsalud.es)

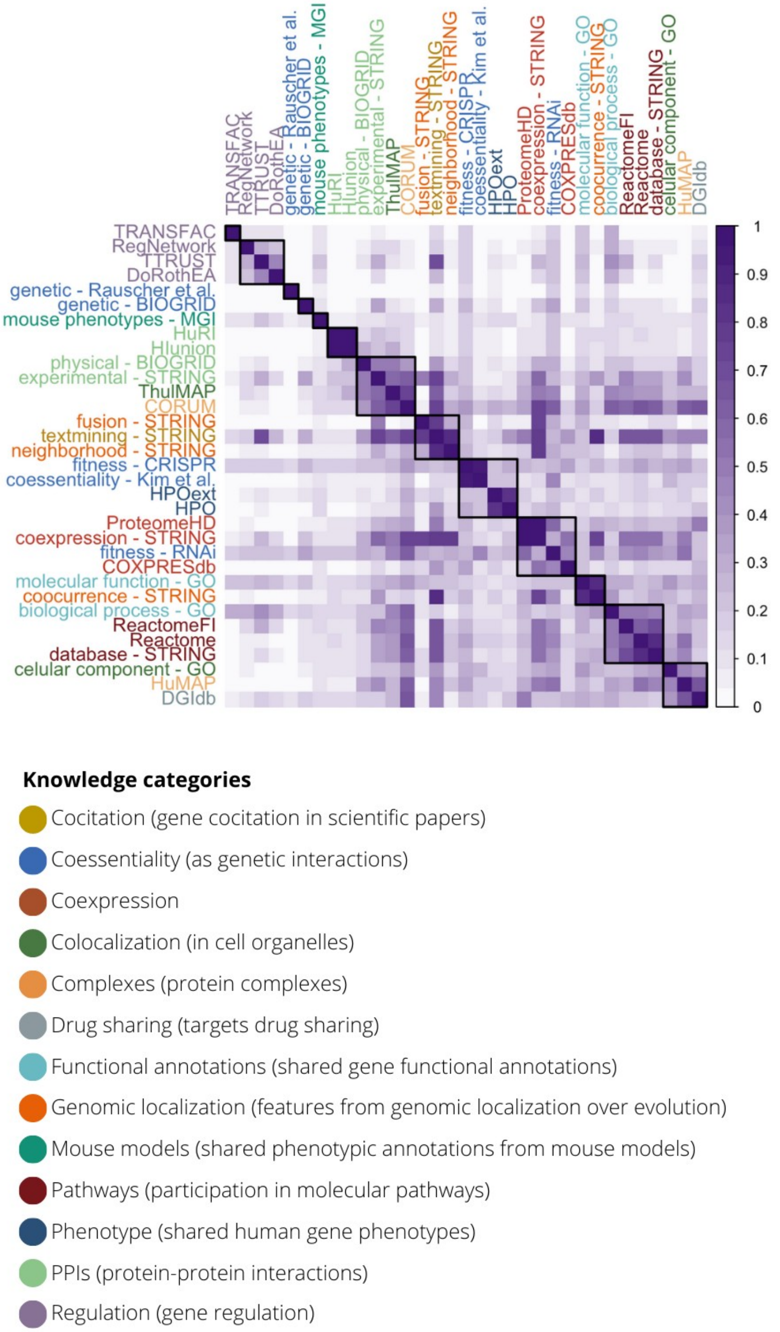

Figure S1. Pairwise network similarity. Edge-wise overlap coefficient is represented for each pair of networks considered in this study. Labels are colored according to the Knowledge Category (KC) in which they are classified. Hierarchical clustering of networks highlights their association by KC and reveals some networks with a global low overlap coefficient as genetic interactions from BIOGRID and, oppositely, shows networks with general high overlap such as textmining from STRING.

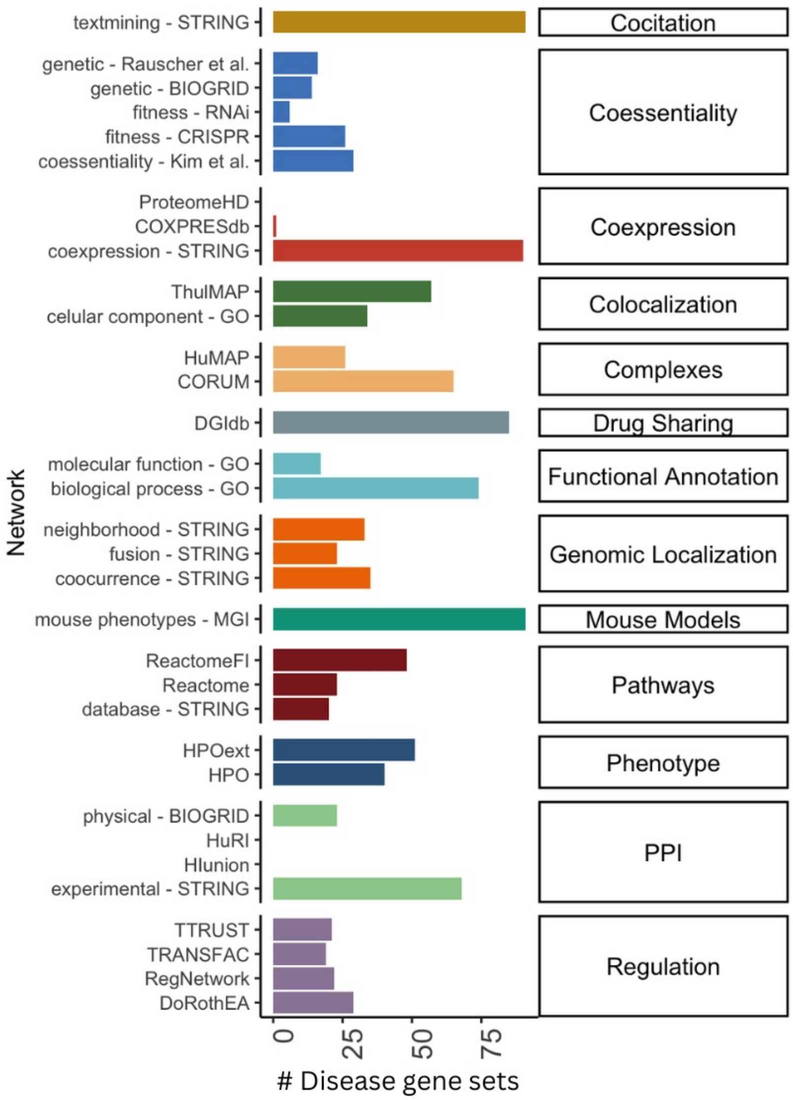

Figure S2: Representative KC network across diseases. For every tested disease gene set (N=91) and knowledge category (KC, N=13), we selected a single individual network based on their ability to prioritized disease genes by using their area under the precision-recall gain (AUPRG) as measure of non-random behaviour. A total of 91 disease genesets were evaluated.

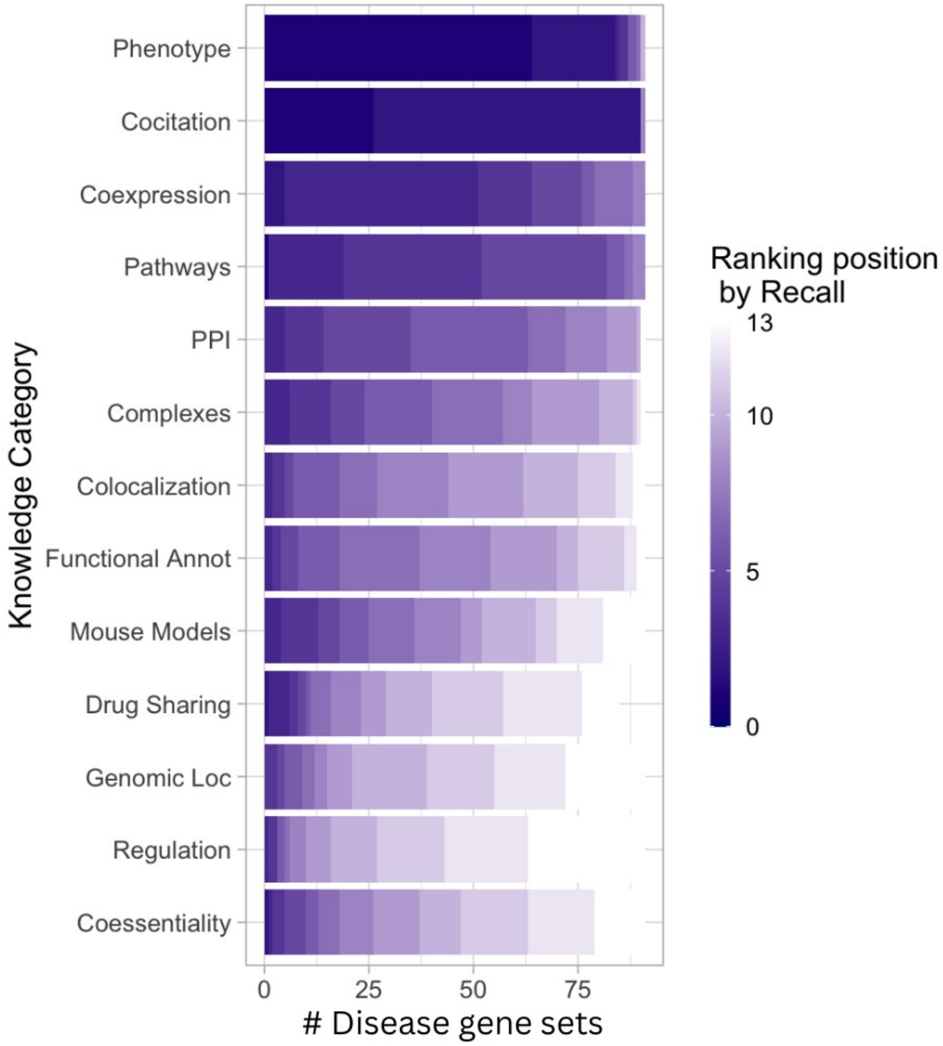

Figure S3: Ranking of KC networks by efficiency in their recovery of gene-disease associations. Recall at n (n = gene set size) was used as efficiency metric.

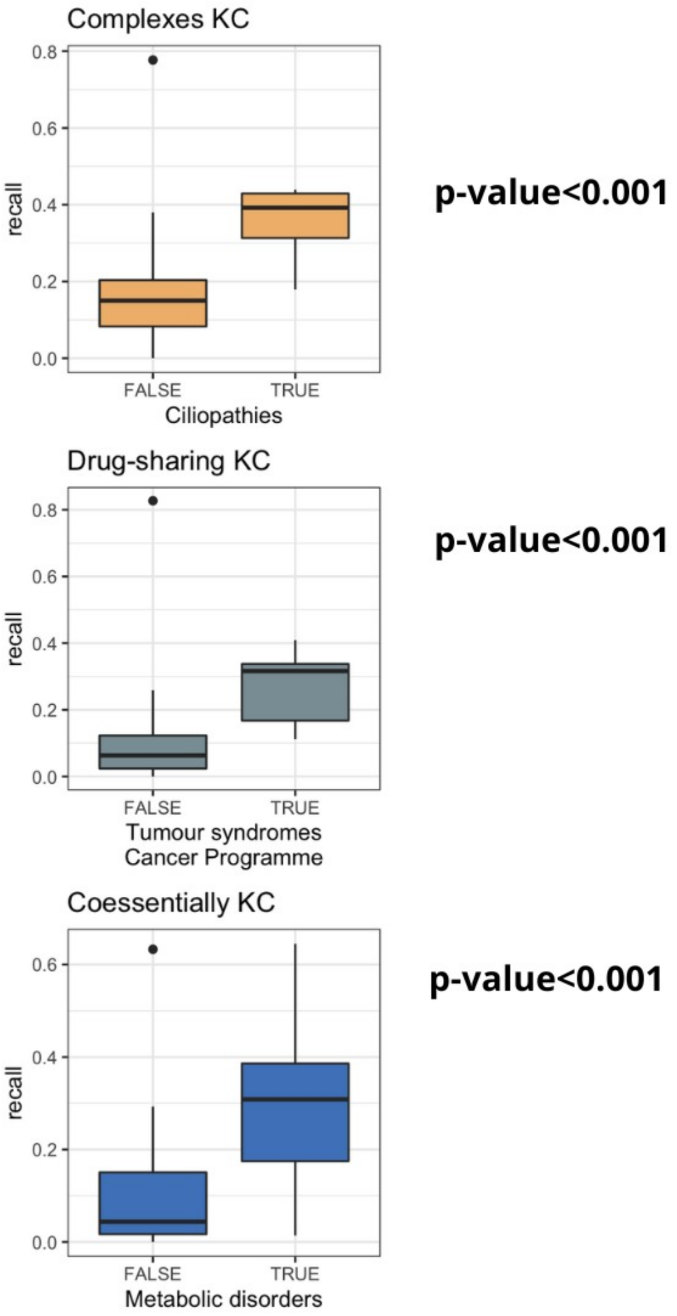

Figure S4: High efficiency of particular KCs in specific disease families. Recall at n-top (n = gene set size) for specific KCs are represented as a distribution classified by: TRUE (diseases of that family) and FALSE (other diseases). Increased efficiency for the disease family under study was tested using the Wilcoxon signed-rank test.

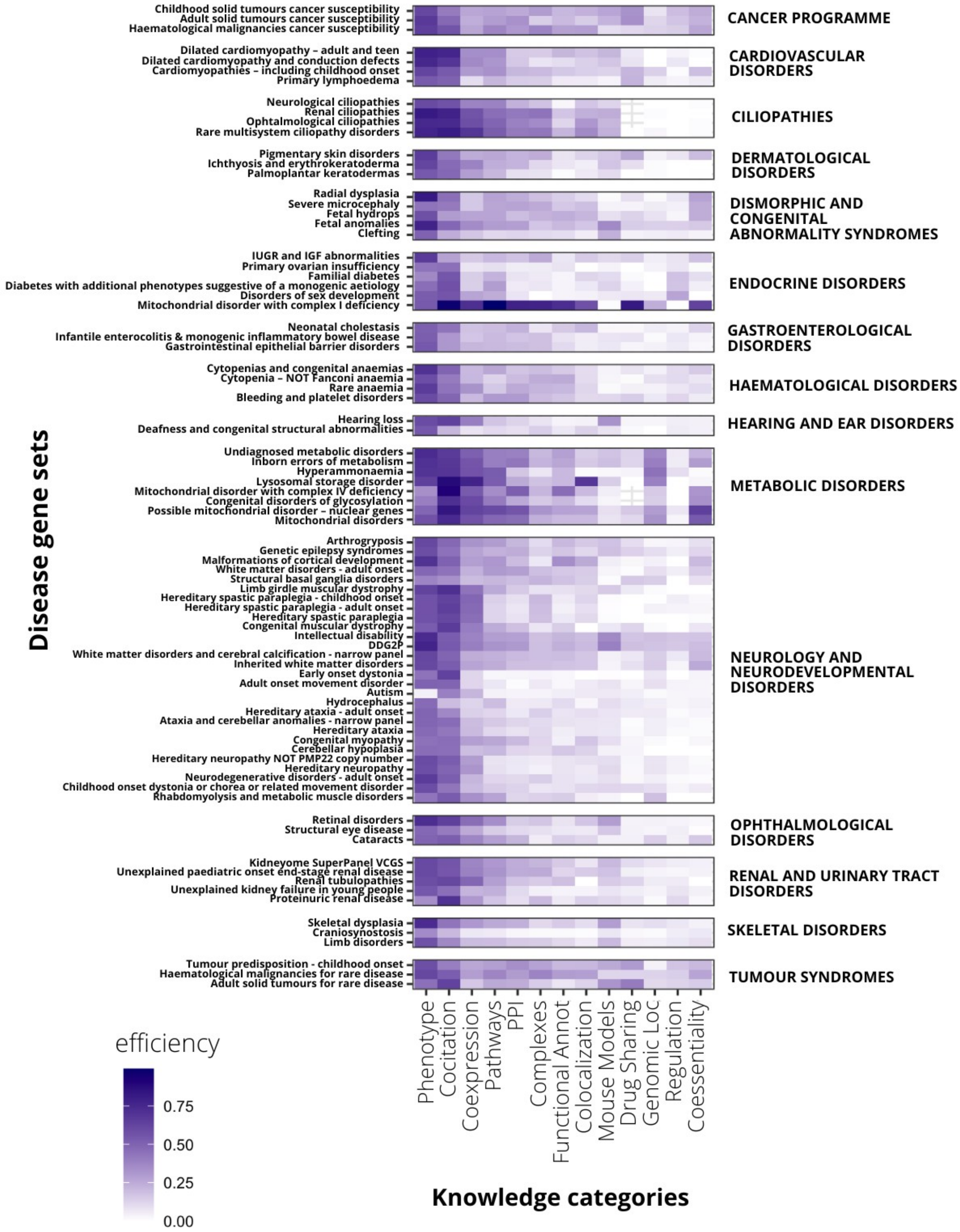

Figure S5. Efficiency in gene recovery for every Knowledge Category (KC) in 91 diseases. Efficiency is measured as recall at n-top (n=number of genes associated to the disease). Diseases are classified in disease families.

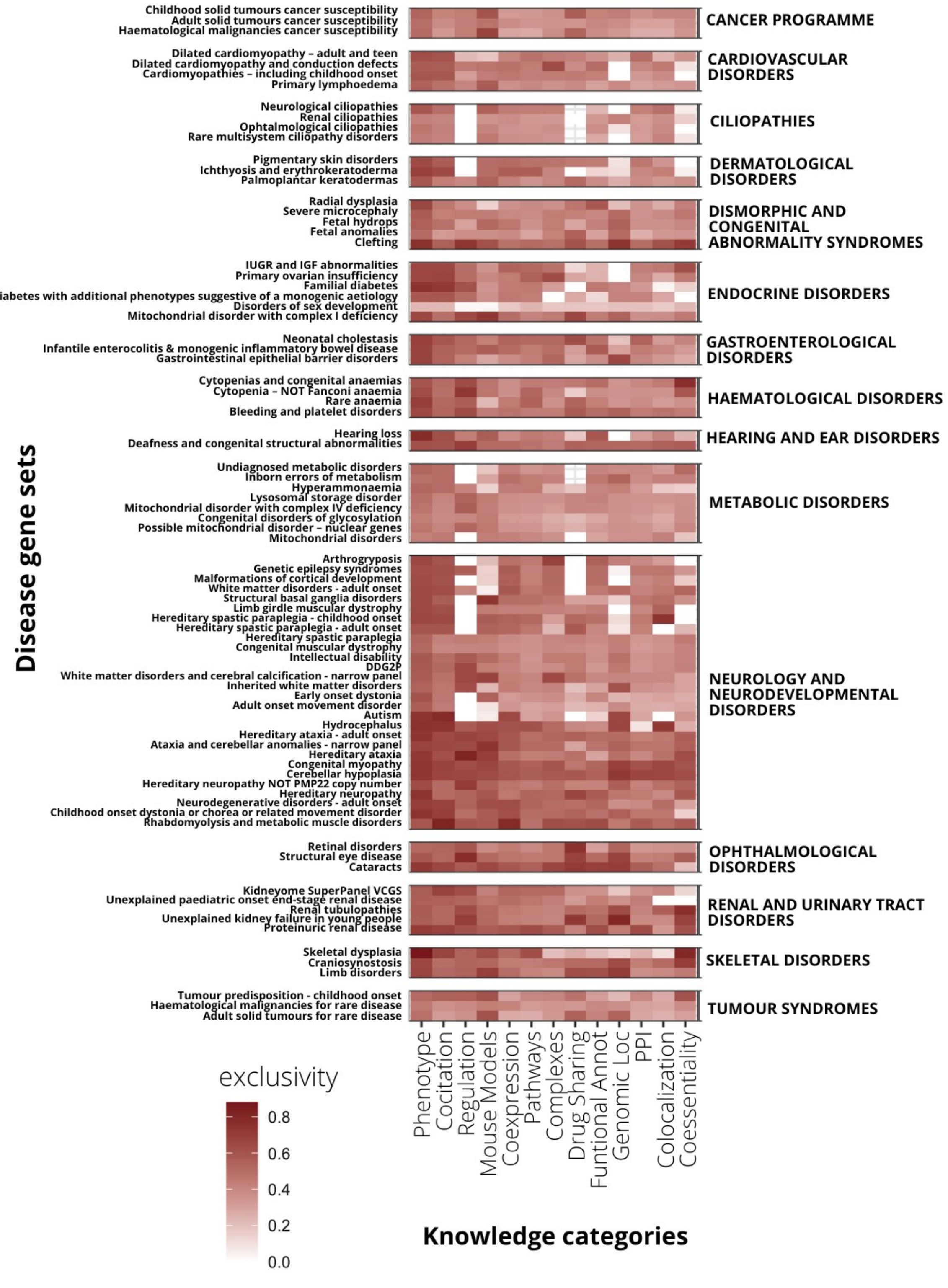

Figure S6. Exclusivity in gene recovery for every Knowledge Category (KC) in 91 diseases. Exclusivity is measured as the average of gene specificity at n-top (n=number of genes associated to the disease). Diseases are classified in disease families.

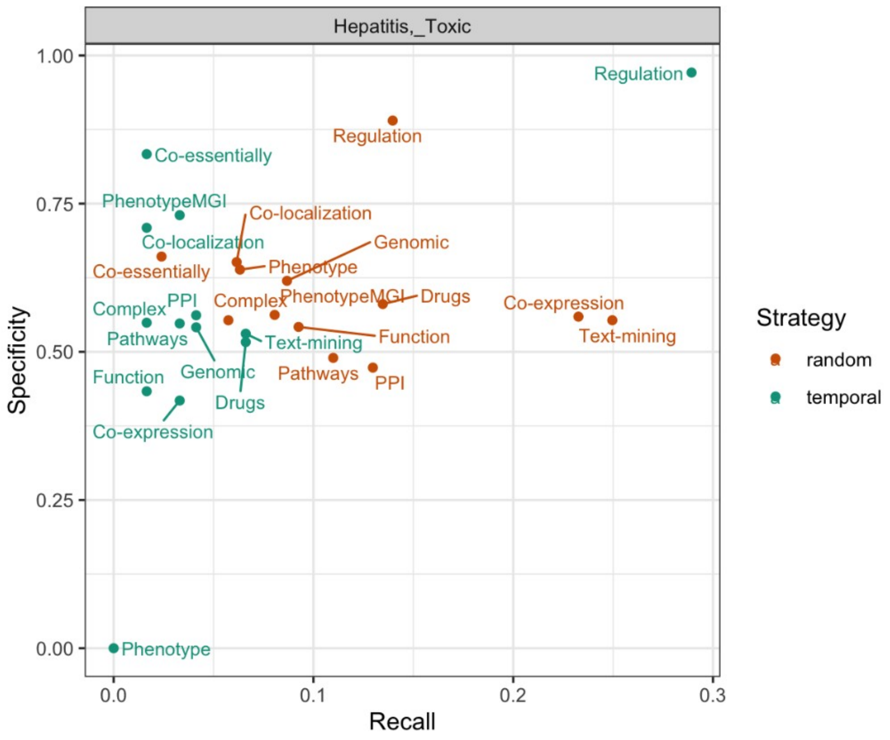

Figure S7. KC performance across alternative network evaluation modes. Scatterplot representing KC specificity and recall relationship for Toxic Hepatitis DisGeNET gene set using both random and time-printed network evaluation.

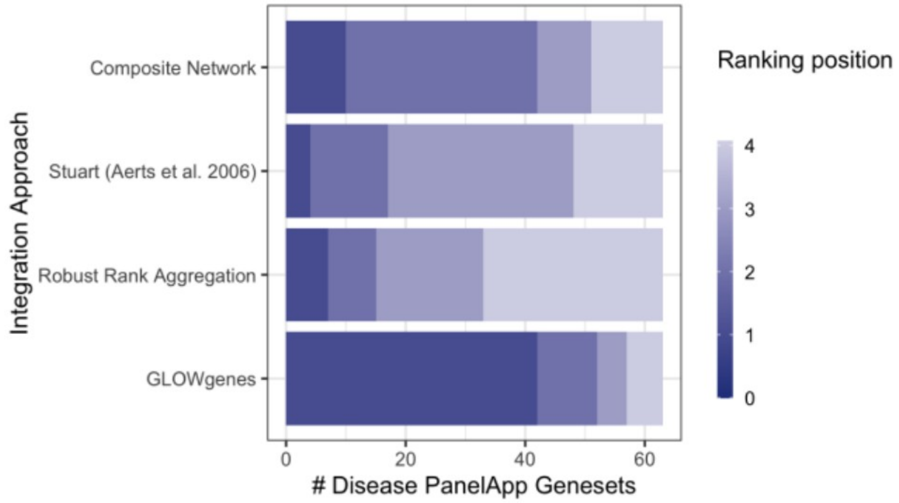

Figure S8: Performance ranking of alternative KC integration approaches across PanelApp disease gene sets at top-n recall (n = size of RG validation set).

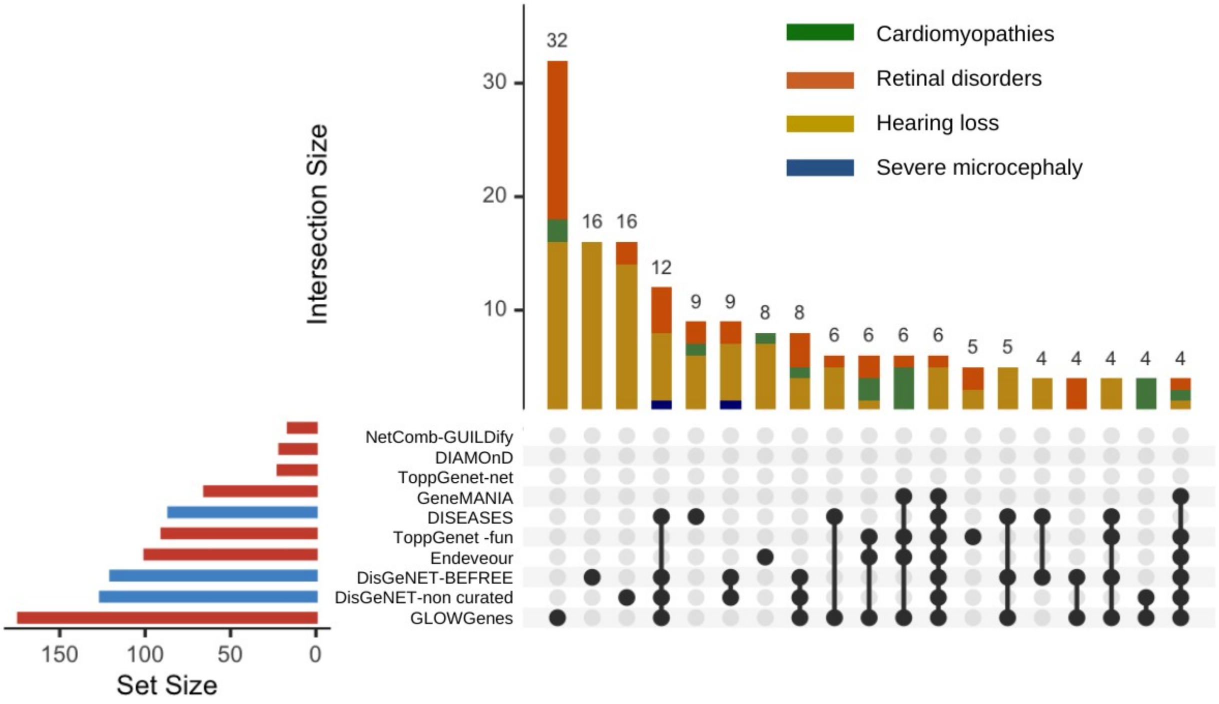

Figure S9. Ranking of methods for prediction of gene-disease associations recovering genes from four diseases. Number and overlap of genes captured by each of the methods evaluated at top-n (n = size of RG validation set). Diseases included are: cardiomyopathies including childhood onset, hearing loss, retinal disorders and severe microcephaly.

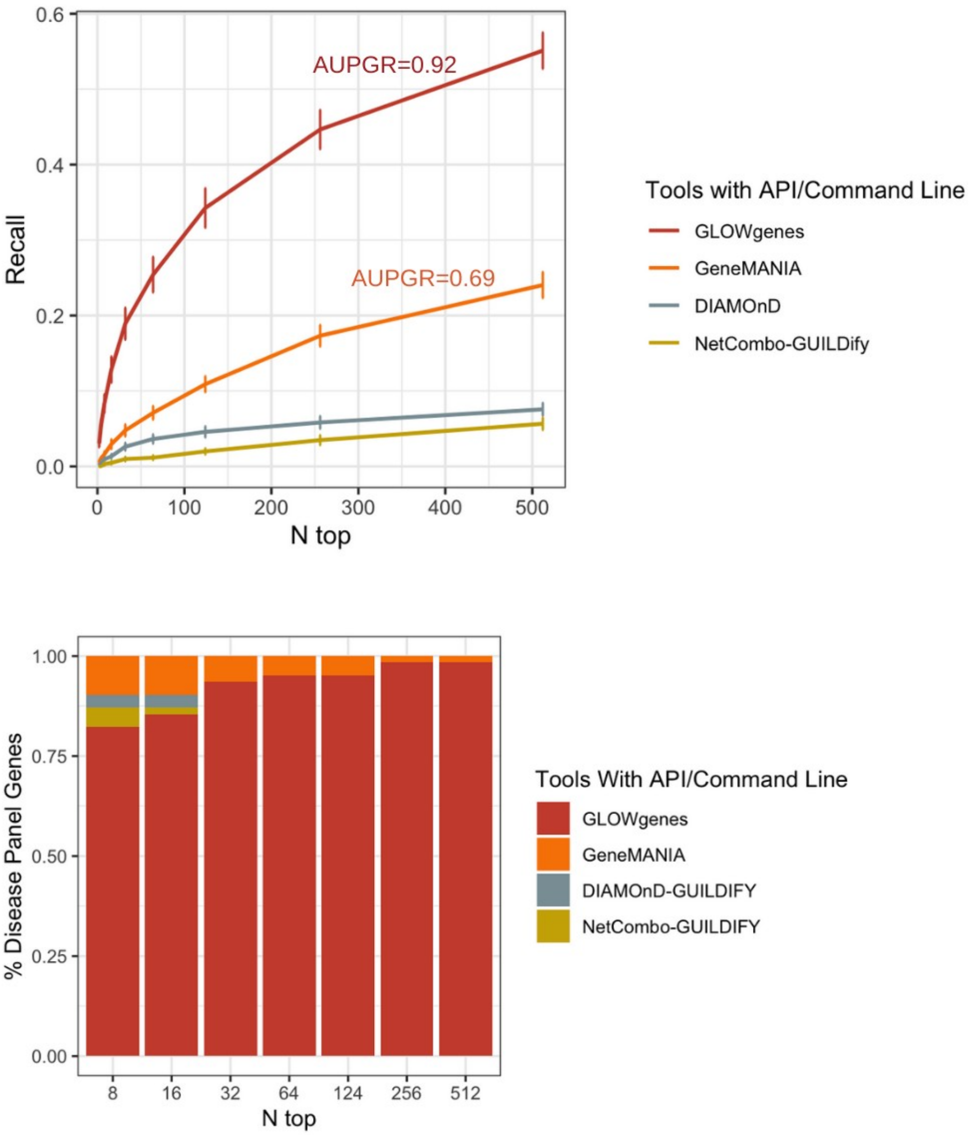

Figure S10. Performance ranking of gene-disease association prediction methods providing programmatic access across 70 PanelApp disease gene sets. Recall at top-n (n = size of RG validation set) was selected as threshold.

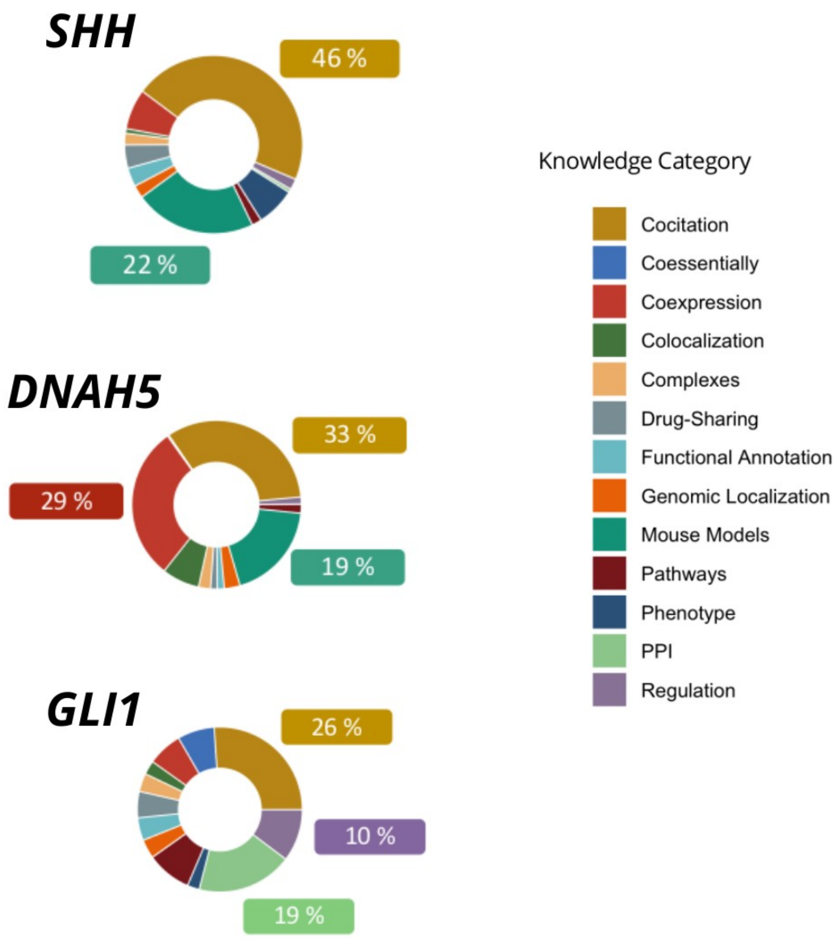

Figure S11. KC contribution in the prediction of three genes (*SHH*, *DNAH5* and *GLI1*) in their association of syndromic inherited retinal dystrophies. Pathogenic variants were found in this three genes in three patients.

Table S1. List of sources with functional information used in this study.

Network	Knowledge Category	Edges weights	Data Source	Download Source	Reference
Textmining - STRING	COCITATION	Textmining score	STRING v11	https://string-db.org/cgi/download.pl	[18]
COXPRESdb	COEXPRESSION	Inverse of the Mutual Rank value	COXPRESdb v7 - Hsa-u.c2-0	https://coxpresdb.jp/download/Hsa-u.c2-0/	[19]
Coexpression - STRING	COEXPRESSION	Coexpression channel score	STRING v11	https://string-db.org/cgi/download.pl	[18]
Coregulation map	COEXPRESSION		ProteomeHD - top-scoring 0.5%	https://www.proteomehd.net/download	[24]
Experimental subcellular map	COLOCALIZATION		Subcellular Localization Experimental Map	http://science.sciencemag.org/highwire/filestream/694217/field_highwire_adjunct_files/9/aal3321_Thul_SM_table_S17.xlsx	[23]
Cellular Component similarity - GO	COLOCALIZATION	Semantic similarity scores	Gene Ontology CC ontology	http://geneontology.org/docs/download-ontology/	[37, 38]
Experimental - STRING	PPI		STRING v11 - experimental channel	https://string-db.org/cgi/download.pl	[18]
Physical PPI - BIOGRID	PPI		BIOGRID 4.2	https://downloads.thebiogrid.org/BioGRID	[25]
HuRI physical PPI	PPI		The Human Reference Interactome	http://www.interactome-atlas.org/	[26]
HI-union physical PPI	PPI		The Human Reference Interactome	http://www.interactome-atlas.org/	[26]
Reactome pathways	PATHWAYS		Reactome	https://reactome.org/download-data	[39]
Reactome FI	PATHWAYS		Pathway-based functional interaction network - v2018	https://reactome.org/about/news/133-reactomefiviz-app-version-7-2-0-released	[28]
Database - STRING	PATHWAYS		STRING v11 - database channel	https://string-db.org/cgi/download.pl	[18]
Molecular function similarity	FUNCTIONAL ANNOTATION	Semantic similarity scores	Gene Ontology MF ontology	http://geneontology.org/docs/download-ontology/	[37, 38]
Biological process similarity	FUNCTIONAL ANNOTATION	Semantic similarity scores	Gene Ontology BP ontology	http://geneontology.org/docs/download-ontology/	[37, 38]
hu.MAP co-complex	COMPLEXES		hu.MAP Experimental Map	http://hu.proteincomplexes.org/download	[31]
CORUM co-complex	COMPLEXES		CORUM	http://mips.helmholtz-muenchen.de/corum/	[36]

				download/ coreComplexes.txt.zip	
Genetic - Rauscher et al.	COESSENTIALITY		Genetic Interaction Network from 85 CRISPR/Cas9 screens in human cancer cells	https://www.embopress.org/doi/full/10.15252/msb.20177656	[27]
Coessentiality - Kim et al.	COESSENTIALITY	weighted co-essentiality	276 high-quality CRISPR knockout screens in cancer cell lines	https://www.ncbi.nlm.nih.gov/pmc/articles/PMC6464042/bin/LSA-2018-00278_TableS5.txt	[29]
Fitness RNAi	COESSENTIALITY	gene fitness rank correlation coefficients	*501 cancer cell lines – RNAi - Achilles Project*	Reproduced following Pan et al. 2018 - https://figshare.com/articles/dataset/Pan_Meyers_et_al_Cell_Systems_2018_/6005297	[35]
Fitness *CRISPR*	COESSENTIALITY		*342 cancer cell lines – CRISPR-Cas - Achilles Project*	Reproduced following Pan et al. 2018 - https://figshare.com/articles/dataset/Pan_Meyers_et_al_Cell_Systems_2018_/6005297	[35]
Genetic - BIOGRID	COESSENTIALITY		BIOGRID 4.2	https://downloads.thebiogrid.org/BioGRID	[25]
DoRothEA regulation	REGULATION	evidence levels transformed into numeric values and normalized to 0-1 range.	DOROTHEA	saezlab.github.io/DoRothEA	[21]
TRANSFAC regulation	REGULATION		TRANSFAC	NDEX	[22]
TTRUST regulation	REGULATION		TTRUSTv2	https://www.grnpedia.org/trrust/	[20]
RegNetwork	REGULATION		RegNetwork	http://www.regnetworkweb.org/	[30]
HPO similarity	PHENOTYPE	Z-scores in the distribution of Jaccard values	HPO similarity	https://hpo.jax.org/app/download/annotation	[32]
Extended HPO similarity	PHENOTYPE	Z-scores in the distribution of Jaccard values	HPO similarity	https://hpo.jax.org/app/download/annotation	[32]
Phenotype - MGI	MOUSE MODELS	Z-scores in the distribution of Jaccard values	MGIphenotype similarity	http://www.informatics.jax.org/downloads/reports/index.html	[33]
DGIdb similarity	DRUG SHARING		DGIdb Drug-Gene interactions	http://www.dgidb.org/downloads	[34]
Neighborhood - STRING	GENOMIC LOCALIZATION		STRING v11- neighborhood channel	https://string-db.org/cgi/download.pl	[18]
Coocurrence - STRING	GENOMIC		STRING v11-	https://string-db.org/cgi/	[18]

	LOCALIZATION		coocurrence channel	download.pl	
Fusion - STRING	GENOMIC LOCALIZATION		STRING v11- fusion channel	https://string-db.org/cgi/download.pl	[18]

Table S2. List of 91 gene sets from the PanelApp resource used to diagnose genetic diseases. Families taken from level 2 in PanelApp classification.

PanelApp disease	Disease family	Number of genes
Childhood solid tumours cancer susceptibility	Cancer programme	82
Adult solid tumours cancer susceptibility	Cancer programme	100
Haematological malignancies cancer susceptibility	Cancer programme	92
Dilated cardiomyopathy – adult and teen	Cardiovascular disorders	46
Dilated cardiomyopathy and conduction defects	Cardiovascular disorders	46
Cardiomyopathies – including childhood onset	Cardiovascular disorders	46
Primary lymphoedema	Cardiovascular disorders	42
Neurological ciliopathies	Ciliopathies	47
Renal ciliopathies	Ciliopathies	76
Ophtalmological ciliopathies	Ciliopathies	61
Rare multisystem ciliopathy disorders	Ciliopathies	99
Pigmentary skin disorders	Dermatological disorders	107
Ichthyosis and erythrokeratoderma	Dermatological disorders	54
Palmoplantar keratodermas	Dermatological disorders	63
Radial dysplasia	Dysmorphic and congenital abnormality syndromes	53
Severe microcephaly	Dysmorphic and congenital abnormality syndromes	106
Fetal hydrops	Dysmorphic and congenital abnormality syndromes	55
Fetal anomalies	Dysmorphic and congenital abnormality syndromes	1326
Clefting	Dysmorphic and congenital abnormality syndromes	188
IUGR and IGF abnormalities	Endocrine disorders	96
Primary ovarian insufficiency	Endocrine disorders	43

Familial diabetes	Endocrine disorders	50
Diabetes with additional phenotypes suggestive of a monogenic aetiology	Endocrine disorders	46
Disorders of sex development	Endocrine disorders	46
Mitochondrial disorder with complex I deficiency	Endocrine disorders	50
Neonatal cholestasis	Gastroenterological disorders	69
Infantile enterocolitis & monogenic inflammatory bowel disease	Gastroenterological disorders	59
Gastrointestinal epithelial barrier disorders	Gastroenterological disorders	54
Cytopenias and congenital anaemias	Haematological disorders	121
Cytopenia – NOT Fanconi anaemia	Haematological disorders	86
Rare anaemia	Haematological disorders	87
Bleeding and platelet disorders	Haematological disorders	109
Hearing loss	Hearing and ear disorders	130
Deafness and congenital structural abnormalities	Hearing and ear disorders	46
Undiagnosed metabolic disorders	Metabolic disorders	677
Inborn errors of metabolism	Metabolic disorders	748
Hyperammonaemia	Metabolic disorders	48
Lysosomal storage disorder	Metabolic disorders	51
Mitochondrial disorder with complex IV deficiency	Metabolic disorders	40
Congenital disorders of glycosylation	Metabolic disorders	84
Possible mitochondrial disorder – nuclear genes	Metabolic disorders	311
Mitochondrial disorders	Metabolic disorders	306
Arthrogryposis	Neurology and neurodevelopmental disorders	141
Genetic epilepsy syndromes	Neurology and neurodevelopmental disorders	575
Malformations of cortical development	Neurology and neurodevelopmental disorders	68
White matter disorders - adult onset	Neurology and neurodevelopmental disorders	77
Structural basal ganglia disorders	Neurology and neurodevelopmental disorders	71
Limb girdle muscular dystrophy	Neurology and neurodevelopmental disorders	168
Hereditary spastic paraplegia -	Neurology and neurodevelopmental disorders	95

childhood onset		
Hereditary spastic paraplegia - adult onset	Neurology and neurodevelopmental disorders	94
Hereditary spastic paraplegia	Neurology and neurodevelopmental disorders	84
Congenital muscular dystrophy	Neurology and neurodevelopmental disorders	44
Intellectual disability	Neurology and neurodevelopmental disorders	1653
DDG2P	Neurology and neurodevelopmental disorders	1673
White matter disorders and cerebral calcification - narrow panel	Neurology and neurodevelopmental disorders	141
Inherited white matter disorders	Neurology and neurodevelopmental disorders	119
Early onset dystonia	Neurology and neurodevelopmental disorders	58
Adult onset movement disorder	Neurology and neurodevelopmental disorders	93
Autism	Neurology and neurodevelopmental disorders	86
Hydrocephalus	Neurology and neurodevelopmental disorders	84
Hereditary ataxia - adult onset	Neurology and neurodevelopmental disorders	201
Ataxia and cerebellar anomalies - narrow panel	Neurology and neurodevelopmental disorders	170
Hereditary ataxia	Neurology and neurodevelopmental disorders	139
Congenital myopathy	Neurology and neurodevelopmental disorders	74
Cerebellar hypoplasia	Neurology and neurodevelopmental disorders	54
Hereditary neuropathy NOT PMP22 copy number	Neurology and neurodevelopmental disorders	175
Hereditary neuropathy	Neurology and neurodevelopmental disorders	186
Neurodegenerative disorders - adult onset	Neurology and neurodevelopmental disorders	144
Childhood onset dystonia or chorea or related movement disorder	Neurology and neurodevelopmental disorders	204
Rhabdomyolysis and metabolic muscle disorders	Neurology and neurodevelopmental disorders	50
Retinal disorders	Ophtalmological disorders	261
Structural eye disease	Ophtalmological disorders	165
Cataracts	Ophtalmological disorders	102
Kidneyome SuperPanel VCGS	Renal and urinary tract disorders	320
Unexplained paediatric onset end-stage renal disease	Renal and urinary tract disorders	181
Renal tubulopathies	Renal and urinary tract disorders	46
Unexplained kidney failure in young people	Renal and urinary tract disorders	84

Proteinuric renal disease	Renal and urinary tract disorders	49
Skeletal dysplasia	Skeletal disorders	406
Craniosynostosis	Skeletal disorders	63
Limb disorders	Skeletal disorders	168
Tumour predisposition - childhood onset	Tumour syndromes	94
Haematological malignancies for rare disease	Tumour syndromes	87
Adult solid tumours for rare disease	Tumour syndromes	58
Ehlers Danlos syndromes	Rheumatological disorders	53
Growth failure in early childhood	No family	48
Laterality disorders and isomerism	No family	43
Primary immunodeficiency	No family	306
Rare genetic inflammatory skin disorders	No family	63

Table S3: List of gene prioritization tools used for comparative assessment.

Name	Strategy	Method	Aggregation Approach	Training Set
**GLOWgenes**	search for genes associated with seeds	Network Based	Integration using ad-hoc network performance	Whole-genome
**Endeavour**	search for genes associated with seeds	Functional similarity	Integration using order statistics	Whole-genome
**ToppGenet - functional similarity**	search for genes associated with seeds	Functional similarity	Statistical meta-analysis (combined p-value)	Neighbours in a network
**ToppGenet - network based**	search for genes associated with seeds	Network Based	Not applied	Neighbours in a network
**NetComb - GUILDify**	search for genes associated with seeds	Network Based	Not applied	Whole-genome
**DIAMOnD**	search for genes associated with seeds	Network Based	Not applied	Whole-genome
**GeneMANIA**	search for genes associated with seeds	Network Based	Composite Functional Association Network	Whole-genome
**DisGeNET - non curated**	pre-defined disease	Gene-disease association	Aggregated Score by number and type of sources	Whole-genome
**DisGeNET - BEFREE**	pre-defined disease	Text-mining	Aggregated Score by number of publications	Whole-genome
**DISEASES**	pre-defined disease	Text-mining	Normalized number of abstracts	Whole-genome

Table S4: Type of evidence sources used by prioritization methods selected for benchmark.

	GLOW genes	Endeavour	ToppGenet - func. similarity	ToppGenet - network based	GUILDify	DIAMOnD	Gene MANIA	DisGeNET-non curated	DisGeNET-BEFREE	DISEASES
**Literature**	x	x	x					x	x	x
**Expression**	x	x	x				x			
**Animal Models**	x		x					x		
**PPI**	x	x	x	x	x	x	x			
**Functional Annotations**	x	x	x							
**Regulation**	x	x	x							
**Genetic Associations**	x	x					x	x		
**Phenotype**	x	x	x				x	x		
**Pathways**	x	x	x				x			
**Genomic Loc.**	x									
**Complexes**	x									
**Chemical information**	x	x								
**Localization**	x	x					x			
**Sequence features**		x					x			
**Interologs**		x					x			

Table S5. Gene set used as virtual panel in the diagnosis of syndromic retinal dystrophies in the Fundacion Jimenez Diaz University Hospital.

ABCA5, ACACB, ACAD9, ACBD5, ADCY3, ALMS1, ANAPC16, AP3D1, AP5M1, AP5Z1, ARHGEF16, ARHGEF17, ARHGEF38, ARL2BP, ARL3, ASIC5, ATP1B2, BICDL2, BPHL, BUG22, C12orf29, c16orf80, CALHM3, CASKIN1, CASZ1, CCDC51, CCP110, CCP110, CEP128, CEP162, CEP164, CEP250, CEP83, CFAP20, CFAP20, CIC, CLUAP1, CNGA3, CNGB3, COBL, COL6A6, COQ8B, COX16, CPNE1, CRTAC1, CWC27, CYP1A1, DACT1, DHX32, DHX34, DIDO1, DMBX1, DMBX1, DPP3, DSCAML1, EDEM3, EFEMP1, EIF4G3, EML4, EP300, EPB41L4A, ERICH6, FAM135B, FAM13A, FAM208B, FAM57B, FBN2, FDFT1, FDXR, FLVCR1, FRMD7, FRMPD2, GMIP, GNB1, GNPTAB, GPR45, GRK1, GRN, GTL3, GUCA1C, HEATR5A, HECTD3, HK1, HSPA9, IFT140, IGSF1, INPP5E, IPO11, IRX1, IRX5, IRX5 - IRX6, IRX6, ITIH2, KCNC2, KCNQ5, KIAA0907, KIF3A, KIF3B, KIFAB3, KIRREL2, LAMA1, LAMB2, LAMC3, LAMP1, LARGE1, LCA5L, Locus arRP, Locus LCA, LTBP1, MAP7D2, MAPRE2, MCM7, METTL9, MFRP, MFSD8, MIGA1, MYO10, MYOM1, NDUFA12, NDUFS3, NUTMD2, NXF1, OTOGL, OTX3, PANK2, PARD3, PARD3B, PCDH15, PCDHGC3, PCM1, PCYT1A, PEX6, PKM, PLA2G5, PLXNB2, PLXNB3, POMGNT2, POMZP3, PPP1R21, PQLC2, PRDM13, PRDM13-IRX1, PREX2, PRPF4B, PRPS1, PTPRN2, PWWP2A, RAB5B, RANBP2, RASGRF2, RCBTB1, RDH12, RECQL4, RGS22, RGS7, RIMS2, RNU4ATAC, ROM1, RP1, RTTN, RUSC1, SAMD7, SAP30, SASS6, SCLT1, SF3B2, SGSH, SH3GL2, SHROOM2, SLC26A7, SLC37A3,
SLC4A7, SLC6A6, SNX29, SON, SSBP1, SUFU, SYTL4, TBC1D32, TBC1D5, TCF20, TECPR2, TJP1,TKTL2, TMED7, TMEM87B, TNXB, TRAPPC14, TRPM2, UBAP1L, USH2A, USP15, USP16, VCAN, VPS13B, VPS13D, VSX2, XPNPEP2
